# Validation of self-reported cardiovascular problems in childhood cancer survivors by contacting general practitioners: feasibility and results

**DOI:** 10.1186/s12875-024-02322-7

**Published:** 2024-03-08

**Authors:** Eva-Maria Hau, Tomáš Sláma, Stefan Essig, Gisela Michel, Laura Wengenroth, Eva Bergstraesser, Nicolas X. von der Weid, Christina Schindera, Claudia E. Kuehni

**Affiliations:** 1 Childhood Cancer Research Group, Institute of Social and Preventive Medicine, University of Bern, Bern, Switzerland; 2 Graduate School for Cellular and Biomedical Sciences, University of Bern, Bern, Switzerland; 3 Center for Primary and Community Care, University of Lucerne, Lucerne, Switzerland; 4 Faculty of Health Sciences and Medicine, University of Lucerne, Lucerne, Switzerland; 5 Institute and Clinic for Occupational, Social and Environmental Medicine, LMU University Hospital, LMU Munich, Munich, Germany; 6 Paediatric Palliative Care and Children’s Research Center CRC, University Children’s Hospital Zurich, Zurich, Switzerland; 7 Division of Paediatric Oncology/Haematology, University Children’s Hospital Basel, University of Basel, Basel, Switzerland; 8 Paediatric Oncology, Inselspital, Bern University Hospital, University of Bern, Bern, Switzerland

**Keywords:** Validation study, Questionnaire, General practitioners, Childhood cancer survivors, Cohort study, Cardiovascular disease, Stroke, Hypertension, Arrhythmia, Thrombosis

## Abstract

**Background:**

Epidemiological studies often rely on self-reported health problems and validation greatly improves study quality. In a study of late effects after childhood cancer, we validated self-reported cardiovascular problems by contacting general practitioners (GPs). This paper describes: (a) the feasibility of this approach; and (b) the agreement between survivor-reports and reports from their GP.

**Methods:**

The Swiss Childhood Cancer Survivor Study (SCCSS) contacts all childhood cancer survivors registered in the Swiss Childhood Cancer Registry since 1976 who survived at least 5 years from cancer diagnosis. We validated answers of all survivors who reported a cardiovascular problem in the questionnaire. Reported cardiovascular problems were hypertension, arrhythmia, congestive heart failure, myocardial infarction, angina pectoris, stroke, thrombosis, and valvular problems. In the questionnaire, we further asked survivors to provide a valid address of their GP and a consent for contact. We sent case-report forms to survivors’ GPs and requested information on cardiovascular diagnoses of their patients. To determine agreement between information reported by survivors and GPs, we calculated Cohen’s kappa (κ) coefficients for each category of cardiovascular problems.

**Results:**

We used questionnaires from 2172 respondents of the SCCSS. Of 290 survivors (13% of 2172) who reported cardiovascular problems, 166 gave consent to contact their GP and provided a valid address. Of those, 135 GPs (81%) replied, and 128 returned the completed case-report form. Survivor-reports were confirmed by 54/128 GPs (42%). Of the 54 GPs, 36 (28% of 128) confirmed the problems as reported by the survivors; 11 (9% of 128) confirmed the reported problem(s) and gave additional information on more cardiovascular outcomes; and seven GPs (5% of 128) confirmed some, but not all cardiovascular problems. Agreement between GPs and survivors was good for stroke (κ = 0.79), moderate for hypertension (κ = 0.51), arrhythmias (κ = 0.41), valvular problems (κ = 0.41) and thrombosis (κ = 0.56), and poor for coronary heart disease (κ = 0.15) and heart failure (κ = 0.32).

**Conclusions:**

Despite excellent GP compliance, it was found unfeasible to validate self-reported cardiovascular problems via GPs because they do not serve as gatekeepers in the Swiss health care system. It is thus necessary to develop other validation methods to improve the quality of patient-reported outcomes.

**Supplementary Information:**

The online version contains supplementary material available at 10.1186/s12875-024-02322-7.

## Background

Collecting accurate information about cardiac late effects among childhood cancer survivors is important because cancer treatments may cause late cardiotoxicity, particularly after treatment with anthracyclines and chest irradiation [1]. Cardiovascular diseases (CVD) are therefore important causes of non-malignant late morbidity and mortality after childhood cancer [2, 3]. Several studies described CVDs in childhood cancer survivors (further referred to as survivors) [4–8], but only two contained validated outcomes: one multi-center [9], and one single-center study [10] validated questionnaire self-reports.

To ensure that information on morbidity is accurate, patient-reported health problems must be validated. Several studies have illustrated problems with the validity of patient-reported cardiovascular diseases [11, 12]. Researchers have suggested different validation methods, including medical assessments [11, 13], use of hospital discharge databases [14] and comparison with medical records from general practitioners (GPs) [11, 12] or hospitals [10, 15, 16].

In the framework of the Swiss Childhood Cancer Survivor Study, we tested a simple and cost-effective approach: validating survivors’ self-reported cardiovascular problems by contacting their GPs. This paper describes (a) the feasibility of this approach; and (b) the agreement between survivor- and GP-reported cardiovascular problems.

## Methods

### Study population

#### The Swiss Childhood Cancer Survivor Study

The population-based Swiss Childhood Cancer Registry (ChCR; www.childhoodcancerregistry.ch) contains information on all children and adolescents in Switzerland who have been diagnosed with cancer since 1976. Inclusion criteria are being diagnosed with leukemia, lymphoma, central nervous system (CNS) tumors, malignant solid tumors, or Langerhans cell histiocytosis at age 0–20 years. The ChCR prospectively collects information on baseline demographics, cancer diagnosis, treatment, and course of disease [17]. The Swiss Childhood Cancer Survivor Study (SCCSS) is a nationwide population-based cohort study that investigates long-term outcomes after childhood cancer [18] including all childhood cancer patients registered in the ChCR who survived five years or more. All participants diagnosed with cancer before age 16 years, who returned the questionnaire and reported a cardiovascular problem, were eligible for this validation study.

### Procedures

#### The Swiss Childhood Cancer Survivor Study (SCCSS) questionnaire survey

Since 2007, the SCCSS sends questionnaires (in German, French or Italian) to all ≥ 5-year survivors. The extensive standardized questionnaire contains questions used in North American and British childhood cancer survivor studies [19–21]. It assesses quality of life, fertility, somatic health, current medication and health service use, psychological distress, health behavior, and socioeconomic information. It also includes a section on current and past cardiovascular health. Adolescent survivors aged 16–19 years at study and parents of survivors under the age of 16 received an age-adapted questionnaire. Non-responders were mailed a second copy of the questionnaire and, if they again failed to respond, were contacted by phone.

#### Survey to general practitioners

All participants in the SCCSS were asked to provide contact details of the GP who knew them best, and to give consent to contact this GP. In Switzerland pediatricians are GPs for all patients under 16 years of age, so we included them in this study. We use the term “GP” for both GPs and primary care pediatricians. In some cases, survivors wrote down hospital-based physicians (mostly pediatric oncologists) instead of GPs. We did not include responses from hospital-based physicians in this analysis.

Validation was done for the questionnaires returned within one data collection wave between 2007 and 2012 (mean time since SCCSS-survey 2.9 years; range 0.3–5.3 years). Each participant contributed with one questionnaire or phone call. All GPs received a study information letter and a case-report form with a pre-paid return envelope (Supplementary File 1) and were asked to transcribe or copy medical records of the survivor’s cardiovascular problems. GPs received no compensation. Those who did not respond within a month were reminded by phone and sent the postal questionnaire again.

### Measurements

Demographic data and information on cancer diagnosis and treatment came from the ChCR and included date of birth, sex, current place of residence, type of cancer, age at diagnosis, and time since diagnosis. Type of cancer was classified by the ChCR according to the International Classification of Childhood Cancer (3rd edition) [22].

### Cardiovascular problems

Information about cardiovascular problems was collected in the survivor questionnaire, and validated against physician reports.

#### Questionnaire to childhood cancer survivors (survivor-reports)

One section of the SCCSS questionnaire assessed survivor-reported health problems, including cardiovascular problems (for details see Supplementary File 2). Survivors were asked: “Have you ever been told by a doctor that you had one of the following diseases?” (1) hypertension, requiring medication; (2) arrhythmias; (3) congestive heart failure; (4) myocardial infarction; (5) angina pectoris; (6) stroke; (7) arteriosclerosis; (8) deep vein thrombosis or pulmonary embolism; (9) valvular problems; (10) other cardiovascular problems; as done in previous studies of survivors [19, 20]. We also asked the year of first diagnosis of the disease.

Each response in the cardiovascular section of the SCCSS questionnaire was coded as ‘yes’ for agreement to having a disease and ‘no’ for disagreement. Answers to closed questions were corrected with information given in ‘free-text comment fields’; for instance, the answers of survivors who wrote ‘cerebral hemorrhage’ were corrected to ‘yes’ for the ‘stroke’ diagnosis category.

#### Survey to general practitioners (validation)

We used an adapted version of the case-report form of the British Childhood Cancer Survivor Study [19] (see Supplementary File 1), and requested information on exact diagnosis, date of diagnosis, diagnostic and therapeutic procedures, and current medication for all cardiovascular problems. We asked the GP for copies of all relevant hospital discharge reports and reports of relevant examinations.

The information from the GP was extracted from the case-report forms and documents received. Cardiovascular problems were then grouped into the same nine categories as in the survivor-reports. We only included GP-reported cardiovascular problems diagnosed before the date of the SCCSS questionnaire completion.

### Statistical analysis

First, we determined the proportions of consenting survivors, GPs with contact details and responding GPs. We further determined the proportion of self-reported cardiovascular problems confirmed by the GP (confirmation rate). For this, we separately assessed the number of survivor-reported problems that were confirmed completely, partly, or not at all by the survivor’s GP.

To determine agreement between information reported by survivors and GPs, we calculated Cohen’s kappa (κ) coefficients with 95%-confidence intervals for each category of cardiovascular problem [23, 24]. Kappa measures inter-rater reliability and captures the extent to which the level of agreement exceeds simple chance: κ ≤ 0.4 indicates poor agreement; κ = 0.41–0.60 moderate agreement; κ = 0.61–0.80 good agreement; and κ ≥ 0.8 excellent agreement [23]. Kappa statistics can be strongly influenced by the prevalence of outcomes in the population and asymmetrical imbalances in marginal totals [25]. Thus we also present the percentage of observed total agreement (number of positive and negative answers by survivors and GPs divided by the total) and the separate proportions of positive and negative agreement (number of answers in positive agreement divided by the average number of positive answers; number of answers in negative agreement divided by the average number of negative answers) [26, 27]. For statistical analyses we used Stata version 16.1 (Stata Corporation, Austin, TX, USA).

## Results

### Prevalence of self-reported cardiovascular problems in childhood cancer survivors

**Fig. 1:**
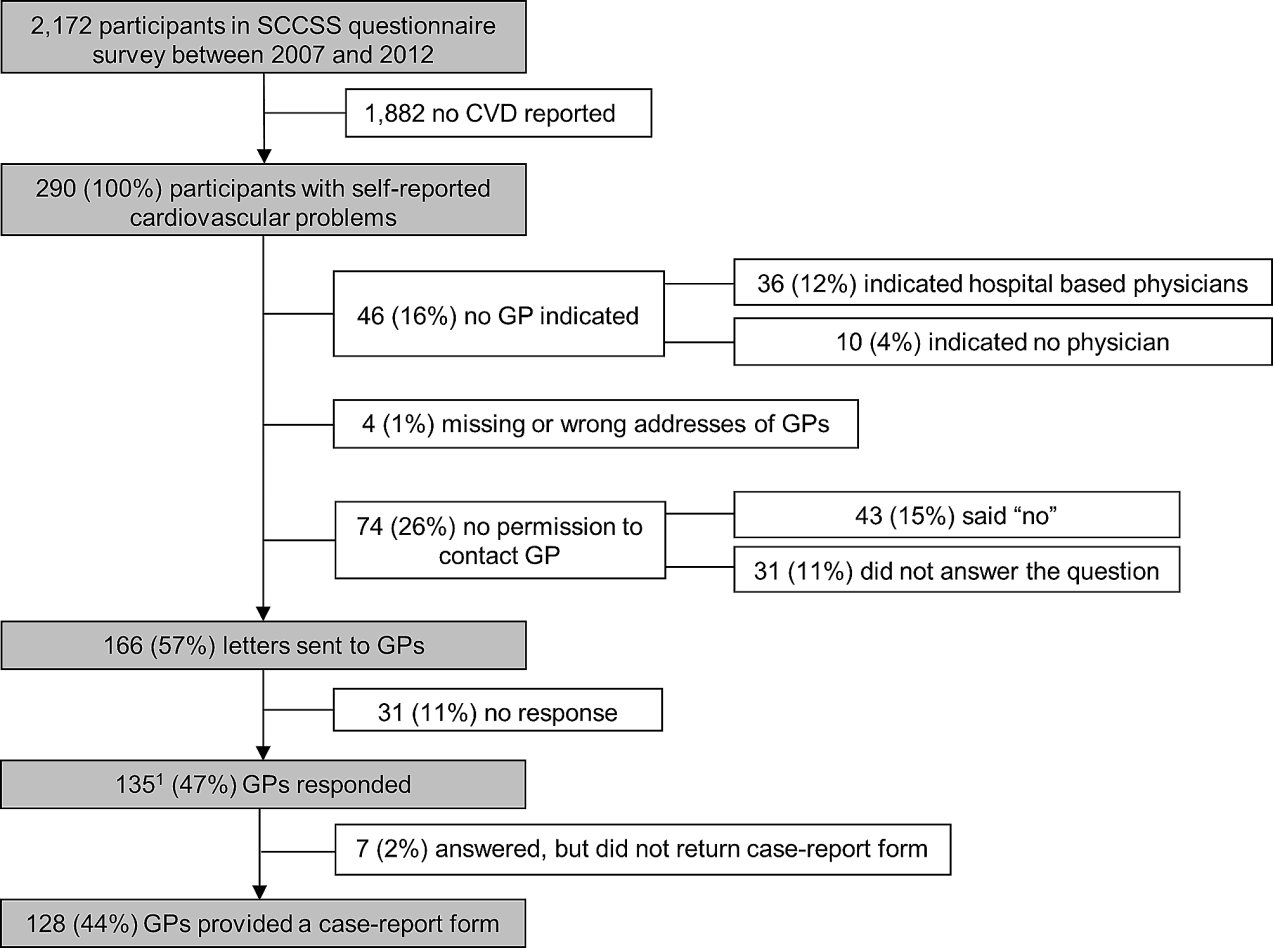
Participants of the Swiss Childhood Cancer Survivor Study and the nested validation study. Abbreviations: GP, general practitioner. ^1^81% of all contacted GPs returned a case report form

By 2012, we had sent questionnaires to 2933 ≥ 5-year survivors. Of those, 2157 returned the questionnaire (Fig. 1). Of those who returned the questionnaire, 290 reported at least one cardiovascular problem and were eligible for the validation study. Reported cardiovascular problems were: (1) hypertension, requiring medication (*n* = 96; 33%); (2) arrhythmias (*n* = 73; 25%); (3) congestive heart failure (*n* = 41; 14%); (4) myocardial infarction (*n* = 2; 1%); (5) angina pectoris (*n* = 23; 8%); (6) stroke (*n* = 7; 2%); (7) deep vein thrombosis or pulmonary embolism (*n* = 23; 8%); (8) valvular problems (*n* = 38; 13%); (9) other cardiovascular problems (*n* = 57; 20%).

Participants reporting a cardiovascular problem were significantly older at study and at cancer diagnosis and had visited more often a GP during the last 12 months (Table 1).

**Table 1:** Characteristics of study population: SCCSS responders without cardiovascular problems vs. responders with cardiovascular problems

	SCCSS responders without cardiac problems (*n* = 1867)		SCCSS responders with cardiac problems^a^ (*n* = 290)		Total (*n* = 2157)		
	*n*	%^b^	*n*	%^b^	*n*	%^b^	*p*-value^c^
*Sex*							0.270
Male	1024	55	149	51	1173	54	
Female	843	45	141	49	984	46	
*Nationality*							0.704
Other	187	10	27	9	214	10	
Swiss	1678	90	263	91	1941	90	
*Language region*							0.286
German	1340	72	217	75	1557	72	
French/Italian	526	28	73	25	599	28	
*Age at study*							< 0.001
< 20y	815	44	76	26	891	41	
20-29y	727	39	134	47	861	40	
30-39y	285	15	60	21	345	16	
40y +	40	2	18	6	58	3	
*Age at diagnosis*							0.001
0–4 years	856	46	111	38	967	45	
5–9 years	492	26	69	24	561	26	
10 years and more	519	28	110	38	629	29	
*Diagnosis (ICCC-3)*							0.013
I Leukemia	661	35	102	35	763	35	
II Lymphoma	285	15	65	22	350	16	
III CNS tumor	282	15	26	9	308	14	
IV Neuroblastoma	97	5	15	5	112	5	
V Retinoblastoma	67	4	6	2	73	3	
VI Renal tumor	130	7	19	7	149	7	
VII Hepatic tumor	16	1	2	1	18	1	
VIII Bone tumor	71	4	16	6	87	4	
IX Soft tissue sarcoma	106	6	12	4	118	6	
X Germ cell tumor	56	3	9	3	65	3	
XI & XII Other tumor^d^	25	1	1	0	26	1	
Langerhans Cell Histiocytosis	71	4	17	6	88	4	
*GP consultations last year*							0.003
no	705	38	83	29	788	37	
yes	1162	62	207	71	1369	64	
*Self-reported cardiovascular problems* ^*e*^							
Hypertension	0	0	96	33	96	5	
Arrhythmia	0	0	73	25	73	3	
Congestive heart failure	0	0	41	14	41	2	
Myocardial infarction	0	0	2	1	2	0	
Coronary heart disease	0	0	23	8	23	1	
Stroke	0	0	7	2	7	0	
Thrombosis/Embolism	0	0	23	8	23	1	
Valvular problems	0	0	38	13	38	2	
Other	0	0	57	20	57	3	

NOTE: Percentages are based upon available data for each variable

Abbreviations: CNS, Central Nervous System; GP, general practitioner; ICCC-3, International Classification of Childhood Cancer - Third Edition; *n*, number; SCCSS, Swiss Childhood Cancer Survivor Study; y, years

^a^ Participants of the Swiss Childhood Cancer Survivor Study (SCCSS) who reported a cardiovascular problem in the questionnaire survey

^b^ Column percentages are given

^c^*P*-value calculated from chi-square statistics (dichotomous variables) or nonparametric trend tests (ordered categorical variables) comparing SCCSS participants with and without cardiovascular problems

^d^ Other malignant epithelial neoplasms, malignant melanomas and other or unspecified malignant neoplasms

^e^ Each participant could have had more than one cardiovascular problem

### Feasibility of obtaining GP reports

For various reasons, we could contact only 166 GPs (57%) for the 290 eligible survivors (Fig. 1). Forty-six survivors did not list a GP (16%), either because they reported that a hospital physician was their caregiver (*n* = 36), or because they did not mention a GP (*n* = 10). Addresses of four GPs (1%) were incorrect or missing. A quarter of the survivors (*n* = 74, 26%) did not give consent, either by saying no (*n* = 43; 15%) to our question about contacting their GP, or by not answering the question (*n* = 31; 11%).

Of the 166 GPs whom we contacted, 135 (46% of 290 survivors) replied, and 31 (11% of 290) did not (Fig. 1). Of the 135 responders, 128 (44% of 290) returned a case-report form, and seven answered but did not return a case-report form. This was because the GP had retired (*n* = 4), the GP did not know the survivor (*n* = 2), or medical records were unavailable (*n* = 1).

### Agreement between survivor- and GP-reported cardiovascular problems

#### Comparison per survivor

**Fig. 2:**
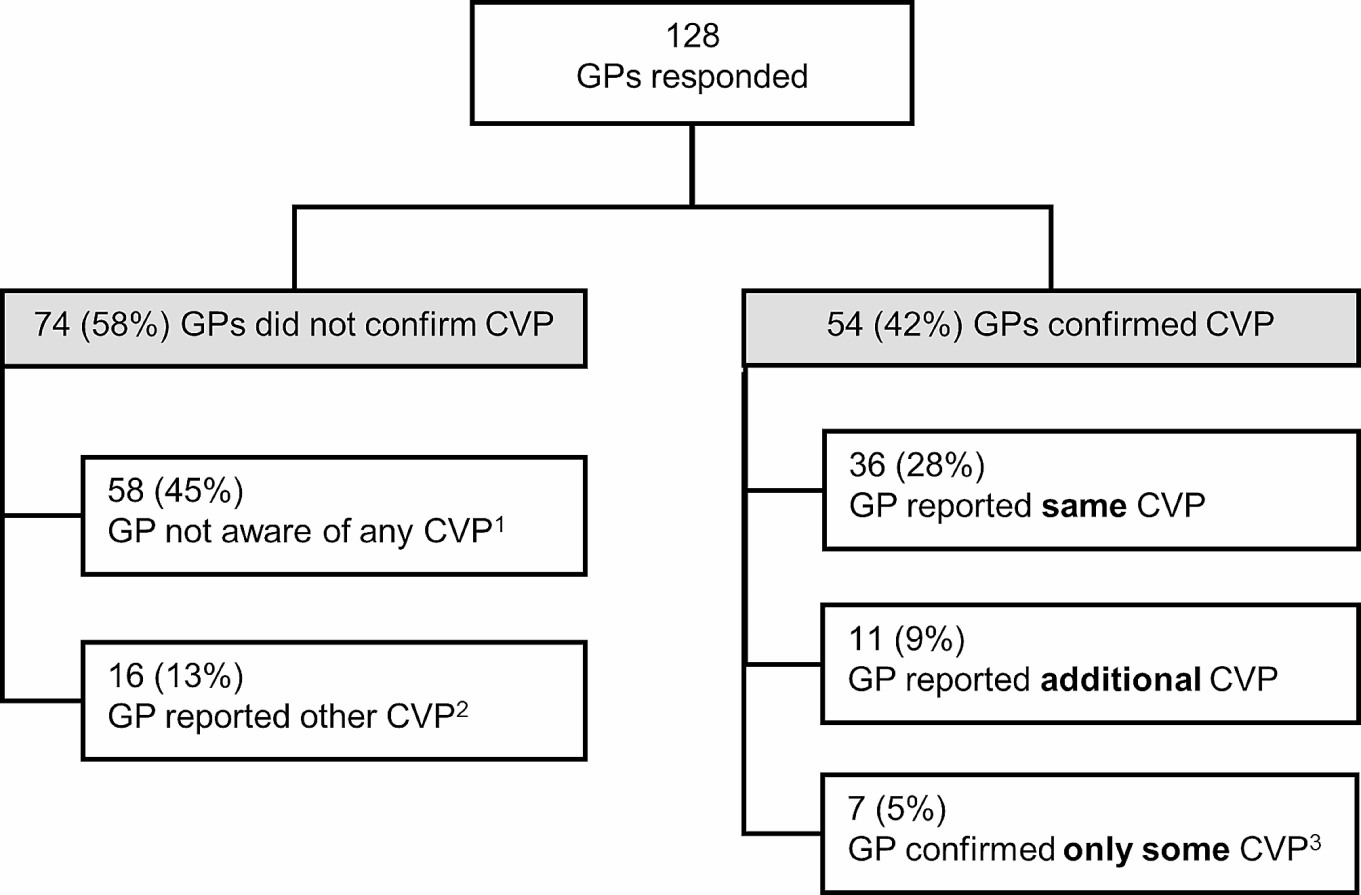
Confirmation of cardiovascular problems by general practitioners. Abbreviations: GP, general practitioner; CVP, cardiovascular problem (one or more). ^1^ Survivor reported a cardiovascular problem, but GP was not aware of any problem. ^2^ GP reported other problems than survivor. ^3^ GP confirmed some, but not all cardiovascular problems reported by the survivor

Of 128 GPs who returned a case-report form, 54 (42%) confirmed that the survivor had a cardiovascular problem (Fig. 2). Of these 54 GPs, 36 (28% of 128) reported the same problems as the survivors; 11 (9% of 128) reported the same problem(s) and gave information on additional problems not reported by the survivor; and seven GPs (5% of 128) confirmed some, but not all cardiovascular problems reported by the survivor.

The remaining 74 of 128 (58%) GPs who returned a case-report form did not confirm the survivor’s self-report (Fig. 2). Of these 74 GPs, 58 (45%) were not aware the survivor had any cardiovascular problem, while 16 (13%) reported that survivors had completely different problems than those indicated in the survey.

#### Comparison per cardiovascular problem

We compared the agreement between survivor-reported problems and GP reports. In total, 152 cardiovascular problems were reported by 128 survivors for whom GP-reports were available (Table 2). Agreement differed between diagnoses and was highest for stroke (98.4%) and lowest for arrhythmias (78.9%). Inter-rater reliability was good for stroke (κ = 0.79; 95% CI, 0.51 to 1.0), moderate for hypertension requiring medication (κ = 0.51; 95% CI 0.33 to 0.69), arrhythmias (κ = 0.41; 95% CI, 0.18 to 0.64), deep vein thrombosis or pulmonary embolism (κ = 0.56; 95% CI, 0.27 to 0.85) and valvular problems (κ = 0.41; 95% CI, 0.18 to 0.64), and poor for angina pectoris (κ = 0.15; 95% CI, -0.08-0.38) and congestive heart failure (κ 0.32; 95% CI, 0.07–0.57). The number of myocardial infarctions was too small (*n* = 2) to be evaluated in our young study population. The discordance in reports of cardiovascular problems was mainly explained by more frequent reporting of problems by survivors – e.g., hypertension was reported by survivors in 17 cases, while it was reported by GP only in 3 cases. Similar patterns were observed across conditions.

**Table 2:** Agreement between answers of general practitioners and survivor’s self-reports (*n* = 128)

	++	+-	-+	--	*P*_o_	*P*_pos_	*P*_neg_	κ^1^	95% CI
Hypertension	15	17	3	93	84.4	60.0	90.3	0.51	(0.33–0.69)
Arrhythmia	15	23	4	86	78.9	52.6	86.4	0.41	(0.24–0.58)
Congestive Heart Failure	5	12	4	107	87.5	38.5	93.0	0.32	(0.07–0.57)
Coronary Heart Disease	2	13	3	110	87.5	20.0	93.2	0.15	(-0.08-0.38)
Stroke	4	0	2	122	98.4	80.0	99.2	0.79	(0.51-1.00)
Thrombosis	5	6	1	116	94.5	58.8	97.1	0.56	(0.27–0.85)
Valvular problems	7	14	2	105	87.5	46.7	92.9	0.41	(0.18–0.64)

Abbreviations: CI, confidence interval; GP, general practitioner; κ, kappa; *n*, number; ++, problem reported by survivor and GP; +-, problem reported by survivor only; -+, problem reported by GP only; --, problem neither reported by GP nor by survivor; *P*_neg_, proportion of observed negative agreement; *P*_0_, proportion of observed total agreement; *P*_pos_, proportion of observed positive agreement; 95% CI, 95% confidence interval of κ

^1^Level of agreement indicated by κ: <0.4 poor, 0.41–0.60 moderate, 0.61–0.80 good, > 0.8 excellent

## Discussion

In this questionnaire survey of childhood cancer survivors in Switzerland, 13% of survivors reported a cardiovascular problem. This study describes the attempt to validate these survivor-reported problems by contacting survivors’ GPs. We were able to contact only 57% of GPs and only 44% of GPs provided information on survivor’s cardiovascular conditions. Overall, the survivor-reported problem was confirmed in only 19% of cases. In many cases, the obstacle to confirmation was the survivor, who either failed to report a GP or denied consent to contact his GP. In contrast, most of the GPs we could contact responded (81%). When they did reply, we found that inter-rater reliability between survivors and GPs varied by diagnosis: it was good for stroke; moderate for hypertension, arrhythmias, valvular problems, and thrombosis; and poor for coronary artery disease, and heart failure.

### Strengths and limitations

To our knowledge this is one of the first studies to evaluate the possibility of validating questionnaire self-reports by contacting GPs in the Swiss health care setting. It is also the first study in Switzerland to focus on validating self-reported cardiovascular problems in childhood cancer survivors. The study was population-based and representative for all childhood cancer survivors in Switzerland.

There are some limitations of this study. First, we did not primarily validate negative reports (instances in which no cardiovascular problem was indicated). However, Olsson et al. found in a population-based study that the number of false negative answers was very small and concluded that validation can be limited to participants with positive replies [16]. Among the negative reports we could validate in our study, the number of “false negative” answers—i.e., survivors did not report problems which were reported by their GP—was also very small. Much more represented were the “false positive” answers—i.e., survivors reported problems which were not confirmed by their GP (Table 2). Second, the questionnaire for survivors relied mainly on closed questions, while the case report forms for GPs used open questions; this may have affected the results. Another reason for non-agreement may be that patients and GPs have different hierarchies concerning what they see as a relevant problem when being asked to answer a question as “What is your problem?“.

### Comparison with other studies

#### Feasibility of obtaining medical reports

Earlier studies were done in countries with different health care systems, so comparison is not straightforward. Most researchers obtained medical records from hospitals or medical registries [28–36]. A few studies validated self-reported cardiovascular diseases directly via GPs as we did [11, 37–42]. Some investigators recruited participants directly from GPs, making identification and contacting GPs not necessary [11, 38–41]. One cataract case-control study in the USA used the same validation procedure as we did and reported a similar physician response rate (86%; ours was 81%) [37]. Mulrooney and colleagues assessed cardiac late effects in the Childhood Cancer Survivor Study (CCSS) in the USA and tried to validate self-reports with medical records from hospitals and GPs [9, 43]. They concluded that this approach was not feasible, since they could not obtain all relevant documents [9, 43].

#### Agreement between survivor- and GP-reports

In our study, agreement was highest for stroke. This is in line with six previous cohort studies showing moderate to good agreement for stroke (κ = 0.43–0.71) [11, 29, 39–42]. Only moderate inter-rater reliability was found in our population for hypertension, thrombosis, valvular problems, and arrhythmias. This is similar to four previous studies, which found moderate agreement between self-reports and physician reports (κ = 0.44–0.56) [40–42, 44]. However, studies combining hospital records and GP-reports for validation found accurate self-reports of hypertension, with good agreement [11, 29]. We found no data on validity of self-reported thrombosis. The German MultiCare Cohort Study found also moderate agreement between self-reported arrhythmias and GP-reports [41]. Finally, we found poor agreement for heart failure and angina pectoris/coronary heart disease. A German hypercholesterinemia cohort study also reported low agreement for angina pectoris (κ = 0.04) and heart failure (κ = 0.29) [11]. Lampe et al. reported good agreement (κ = 0.72) between self-reported angina pectoris and GP-reports [38]. Kehoe described low sensitivity (64%) but relatively high specificity (96%) for self-reported coronary heart diseases [37]. A study on heart failure among > 45 year old Minnesota residents found moderate agreement between self-report and medical record (κ = 0.46) [29].

#### Validation of self-reported cardiovascular problems in childhood cancer survivors

To our knowledge, only two studies published validated questionnaire self-reports on cardiovascular problems in childhood cancer survivors [9, 10]. Among participants for whom medical report validation was successful, the CCSS has successfully confirmed congestive heart failure in 67% [9]. However, medical report validation could be performed in only 35% of survivors who self-reported the outcome, hence the authors concluded it was not feasible to utilize outcomes validated this way [9]. A study of bone marrow transplanted survivors in the USA validated survivor-reports and found good to excellent agreement between self-reports of cardiovascular diseases and medical records (κ = 0.7–0.8) [10]. However, they validated only myocardial infarction, hypertension, and overall cardiac events. The discrepancy between our findings and theirs might be explained by differences in study populations and methods. They assessed agreement between self-reported complications and medical records for only the first 100 respondents. It is possible that early respondents are more motivated survivors (with special interest in late effects) and answer differently than late responders. Second, their study participants all came from the same highly specialized pediatric oncology center, and all had a bone marrow transplantation. This might have influenced their results since these survivors are usually involved in strict follow-up programs and might thereby be better informed.

### Interpretation of the results

#### Feasibility of obtaining medical reports

Although the response rate of GPs was excellent (81%), our validation method failed for several reasons. First, many survivors did not consent to contact GPs or did not provide sufficient contact details. Second, many survivors did not have a GP, but listed their former pediatric oncologist as the person who knew their current health problems best. This indicates that the survivor might not have been referred back to primary care or might not have gone there. Third, some GPs had retired, or did not have records available.

#### Agreement between survivors and GPs

The agreement was low for two probable reasons: inaccurate reporting by the survivor or incorrect reporting by the GP. *Inaccurate reporting by survivors* could be caused by poor understanding of their health problem. Self-reports are most accurate for well-defined diseases with clear symptomatology like thrombosis, hypertension, myocardial infarction and stroke, which is easy to understand for patients [37, 45, 46]. Our findings underline this: we found lower agreement for poorly defined diseases with a wide spectrum of symptoms and causes, like arrhythmias, heart failure and coronary heart disease. Single high-impact events such as strokes are well reported since they result in hospitalization and patients do not forget them. Moreover, the neurological symptoms of a stroke often persist long after initial hospitalization and patients are reminded of this event in their everyday life. As for CVD with moderate agreement (e.g., hypertension, arrhythmia), previous studies found that awareness of these conditions depends on length of treatment duration, employment, and education status [47, 48]. Coronary artery disease and heart failure—CVD with poor agreement—can vary dramatically in their presentation over time, which determines the patients’ evaluation of their illness, their self-care and thus their GP’s knowledge of the diagnosis. *Inaccurate reporting by GPs* can result if the GP is unaware of the cardiovascular problem. In the Swiss health care system, survivors can see several doctors in parallel and GPs are usually not gate-keepers to specialized healthcare [49]. Therefore, survivors may have consulted different doctors for their cardiovascular problems, and their GP may not have been informed.

### Follow-up care in the Swiss health care system

In Switzerland, children diagnosed with cancer are treated in one of nine pediatric oncology clinics. After completion of treatment, survivors are followed-up at the treatment center for up to 10 years or until the age of 18 years. Many are then transferred back to the primary care system or specialists such as endocrinologists or oncologists depending on the individual’s outcome. This transfer is often complicated [50, 51]. Even if survivors choose a GP for follow-up care, their GP might not be aware of all their health problems. A recent survey among Swiss GPs showed, that 74% of respondents were not aware of survivors needing follow-up, and 28% also stated that they do not have enough experience to provide follow-up care [51]. Survivors transitioning to a GP may therefore receive insufficient follow-up care due to the current state of awareness among GPs.

### Implications for practice

In Switzerland, it is not feasible to validate patient-reported cardiovascular events via GPs since the information flow between primary care providers and specialists or tertiary care centers seems to be inadequate, so alternative methods must be attempted. To validate self-reported events, medical examinations of survivors might be performed, or their data linked with data from diagnostic registries or hospital databases. For clinical purposes, information flow between health care providers might also be improved by the electronic patient dossier of the Swiss Federal Office of Public Health. Its introduction is currently being revised [52]. Another way of improving the flow of information between primary care providers and specialists or tertiary care centers would be to increase the proportion of those registered with a GP. This could be achieved by introducing incentives for patients (e.g., lowering health insurance charges) or providers (e.g., capitation payments), or by mandatory registration [53]. As a result, the gatekeeping function of GPs would be strengthened.

Long-term follow-up (LTFU) programs for childhood cancer survivors in Switzerland may provide easier access to information on late effects. In such a program (e.g., using Passport for Care®), follow-up clinics serve as gatekeepers and maintain information from multiple disciplines [54]. Until now, only a small proportion of adult survivors is served by a LTFU clinic in Switzerland [55].

## Conclusions

Despite excellent GP compliance, it was not feasible to validate self-reported cardiovascular problems via GPs because they do not serve as gatekeepers in the Swiss health care system. It is thus necessary to develop other validation methods to improve the quality of patient-reported outcomes, and to introduce policies improving the flow of information between health care providers. This would be beneficial not only for research purposes, but more importantly for a better clinical care of patients.

### Electronic supplementary material

Below is the link to the electronic supplementary material.

Supplementary Material 1

Supplementary Material 2

## Data Availability

The data generated and analyzed during the current study are not publicly available to maintain the privacy of participants. Data is available from the corresponding author upon reasonable request.

## Abbreviations

CCSS: Childhood Cancer Survivor Study
CVD: Cardiovascular diseases
CNS: Central nervous system
GP: General practitioners
LTFU: Long-term follow-up
ChCR: Swiss Childhood Cancer Registry
SCCSS: Swiss Childhood Cancer Survivor Study
USA: United States of America
