# Supplementary material for: Validation of self-reported cardiovascular problems in childhood cancer survivors by contacting general practitioners: feasibility and results
Source: BMC Prim Care. 2024 Mar 8;25:81. doi: 10.1186/s12875-024-02322-7 (PMC10921568; doi:10.1186/s12875-024-02322-7)
Supplement: Supplementary file 1 — Supplementary Material 1 [file 12875_2024_2322_MOESM1_ESM.docx]

## **Supplementary File 1: Case Report Form for General Practitioners**

**CARDIOVASCULAR CONDITIONS/PROCEDURES CONFIRMATION SHEET**

Confirmation from the General Practitioner

| **Surname, name** |
| --- |
| **Date of birth** |

**Please specify below any cardiovascular conditions or procedures ever experienced by this patient:**

| **CARDIOVASCULAR CONDITION**  (e.g., acute coronary syndrome/ myocardial infarction, cardiomyopathy, heart failure, hypertension, arrhythmia, valvular problems, peripheral artery occlusive disease, pericarditis, myocarditis, thrombosis, pulmonary embolism, stroke, etc.) | | **Date initially diagnosed** |
| --- | --- | --- |
| 1 |  | / / |
| 2 |  | / / |
| 3 |  | / / |
| 4 |  | / / |
| 5 |  | / / |

| **CARDIOVASCULAR PROCEDURE**  (Eg. heart catheterisation, angioplasty or coronary artery bypass surgery, valvular surgery, heart transplantation, other heart surgeries, etc.) | | **Date of procedure** |
| --- | --- | --- |
| 1 |  | / / |
| 2 |  | / / |
| 3 |  | / / |
| 4 |  | / / |

| **Current Medication**  (e.g. diuretics, beta blocker, anti-platelet drug, calcium channel blocker, statins, ACE-inhibitors, AT2-antagonists, glycosides, antiarrhythmic medication, nitrates, other antihypertensive medication, oral anticoagulants, heparin, etc…) | |
| --- | --- |
| 1 |  |
| 2 |  |
| 3 |  |
| 4 |  |

**Please provide us with a copy of any of the following medical records:**

- **Any relevant hospital discharge reports**
- **Reports of relevant tests/examinations, e.g.:**

Last ECG

Echocardiography

Cardiac stress test

Holter-monitor

Angiography

CT/MRI

Relevant blood tests (BNP)

- **Any other relevant written communication**

**Has your patient ever been assessed by a cardiologist, or other specialist, in relation to a cardiovascular condition?**

| **Yes** |  |  | **No** |  |
| --- | --- | --- | --- | --- |

**If yes, please provide the following details:**

| Consultant Name: | Consultant Name: |
| --- | --- |
|  |  |
| Hospital name & address: | Hospital name & address: |
|  |  |

**Thank you for your help.**

**Please return this form together with copies of confirmatory clinical letters/reports* with this Pre-paid envelope.**

| Signature: | …………………………………………………… | Stamp |  |
| --- | --- | --- | --- |
|  |  |  |  |
| Printed Name: | …………………………………………………… | Date completed: | ………………………………………………… |
