# Supplementary material for: Validation of self-reported cardiovascular problems in childhood cancer survivors by contacting general practitioners: feasibility and results
Source: BMC Prim Care. 2024 Mar 8;25:81. doi: 10.1186/s12875-024-02322-7 (PMC10921568; doi:10.1186/s12875-024-02322-7)
Supplement: Supplementary file 2 — Supplementary Material 2 [file 12875_2024_2322_MOESM2_ESM.docx]

## **Supplementary File 2: Extraction from the SCCSS questionnaire**

| *The following questions refer to medical conditions which have ever occurred over the course of your life*.  - Please indicate **whether you have ever been told by a doctor that you have any of the following problems**.  - Please indicate **when** the medical condition first occurred and whether **it is still ongoing.**  - Please do not leave any questions unanswered: mark ‚no’ if you have never had the problem. |
| --- |

| **Heart and Circulatory System** |
| --- |

|  | **Ever occurred?** | | **Since when?** | **Still ongoing?** | | |
| --- | --- | --- | --- | --- | --- | --- |
|  | **Yes** | **No** |  | **Yes** | **No** |  |
| Hypertension (high blood pressure), requiring medication |  |  | (year) |  |  |  |
| Irregular heartbeat or palpitations (Arrhythmia), requiring medication or follow-up by a physician |  |  | (year) |  |  |  |
| Congestive heart failure (weak heart muscle) |  |  | (year) |  |  |  |
| A myocardial infarction (heart attack) |  |  | (year) |  |  |  |
| Angina pectoris (chest pains due to lack of oxygen to heart requiring medication such as nitroglycerine) |  |  | (year) |  |  |  |
| A stroke |  |  | (year) |  |  |  |
| Hardening of the arteries or arteriosclerosis |  |  | (year) |  |  |  |
| Deep vein thrombosis or pulmonary embolism |  |  | (year) |  |  |  |
| Stiff or leaking heart valves |  |  | (year) |  |  |  |
| Have you ever had an examination performed by a cardiologist, e.g. a biopsy or a catheter examination? |  |  | (year) |  |  |  |
| Any other heart or circulatory problem  **If Yes**, please describe the problem  _______________________________________________  _______________________________________________ |  |  | (year) |  |  |  |
